# Supplementary material for: Patterns of Intron Gain and Loss in Fungi
Source: PLoS Biol. 2004 Nov 30;2(12):e422. doi: 10.1371/journal.pbio.0020422 (PMC532390; doi:10.1371/journal.pbio.0020422)
Supplement: Table S1 — Also available at http://genes.mit.edu/NielsenEtAl/. (4.3 MB ZIP). [file pbio.0020422.st001.zip › NielsenEtAl/html/1367.html]

AN4282.1.NCU02319.1.MG09190.1.FG01604.1


```
 CLUSTAL W (1.82) Multiple Sequence Alignments - Introns Inserted


Sequence 1: NCU02319.1	1059 aa
Sequence 2: MG09190.1	890 aa
Sequence 3: FG01604.1	984 aa
Sequence 4: AN4282.1	879 aa
Alignment Length: 1073 aa
Number Identitical Residues: 359 aa
Alignment Score (without introns) 20308


MG09190.1 	------------------------------------------------------------
NCU02319.1	MHLAAVRTKTNAVAIGRWKTRSLSTTTTTTTTTTPLSRKSSSPRTIQAIIAAGARPSCTR
FG01604.1 	--------------------------------------MSSSMGLLRHLQAQQQR-----
AN4282.1  	------------------------------------------------------------
          	                                                            

MG09190.1 	------------------------------------------------------------
NCU02319.1	IAASRPLLASSSSSSFSSSSTPASFVIRPSSVTTSTTQHVSRFHSVSSSLLSVPSSSSSS
FG01604.1 	---------------LWSCCSPLSVITRQTSAAAAS-------ASVPQGIFNGIGFGFAR
AN4282.1  	------------------------------------------------------------
          	                                                            

MG09190.1 	------------------------------~-------------------------MSAR
NCU02319.1	SSTSTSTSATSSPRTSTFTTSRLRPKLQTC~AVPPPFLVLGSRYCSAHARAKRRENMTDR
FG01604.1 	GLTSLVNGG--QPLSNRRDNGRPMPIGIRS2LVP----KISDRACSAHARLARNETMTDR
AN4282.1  	------------------------------~-----------------------MASKDR
          	                                                         . *

MG09190.1 	DILPDN~FRPVNYDLQLTDLDFD-NWSYNGTVE2ISGELTKPTTEIVLNVLEIKLKEAQL
NCU02319.1	DILPDN~FKPIHYDLEIRDLDFT-SWSYKGTVR2IDGKLVKPTKDIVLNTLEIKLLNAKL
FG01604.1 	DVLPDN~VKPKHYQLSLRDLEFT-NWTYKGTVT2IDSEITKPTKEIIVNTLELKLSHAKV
AN4282.1  	DILPNV2VKPAHYDISLFDLQLGGSWSYKGIVN~ITSKVYSPTREVVLNAKEIEVHHAKV
          	*:**:  .:* :*::.: **:: ..*:*:* *  * .:: .** ::::*. *::: .*::

MG09190.1 	EIQ0GTKATV~SQSTQNFTYNDKQQRATMVFEEEIPPSAKASLKIKFSGLINHDMAGFYR
NCU02319.1	TAG~----PQ~SWESTQFAEDTKAQRSTISFAEELPVADNVSLTLDFTGELNHDMAGFYR
FG01604.1 	FVD~----SK~LVESTKFDYDAKAQRSTISFDEELPVASKAVITIEFEGIINNEMAGFYR
AN4282.1  	LGL~----DE1LTKTSEITYDQKSERVTIRFPKELPQSE-LVVSISFTGTMNNAMAGFYR
          	             .: ::  : * :* *: * :*:* :    :.:.* * :*: ******

MG09190.1 	SMYKPAVPAAASVPRDEQFHYMLSTQFEACDARRAFPCFDEPNLKATFDIAIEIPSDQVA
NCU02319.1	SQYKPAAPAAASVPRDDEFHYMLSTQFESCDARRAFPCFDEPNLKATFDFAIEIPDDQVA
FG01604.1 	SKYKPTGTPSASVPDDGEWHYMFSTQFEACDARRAFPCFDEPNLKATFDFDIEIPSDQVA
AN4282.1  	SKYKPAVEPTSDTPKEGEFYYMLSTQFESCDARRAFPCFDEPNLKATFDFEIEVPKGQTA
          	* ***:  .::..* : :::**:*****:********************: **:*..*.*

MG09190.1 	LSNMPVKESKE-TAPGKTLVSFDRTPVMSTY~LVAWAVGDFEYIEAFTERRYNGKQLPVR
NCU02319.1	LSNMPVKETKP-AGPNKKLVSFERSPVMSTY~LLAWAVGDFEYVEAFTDREYNGKKLPVR
FG01604.1 	LSNMPVKETRP-SKDGWNIVSFETSPVMSTY~LLAWAVGDFEYIEAFTDRKYNGKQIPVR
AN4282.1  	ISNMPVKSEREGSKPELKVVSFDTTPVMSTY0LLAWAIGDFDYVEALTERKYQGKSIPVR
          	:******. : .:    .:***: :****** *:***:***:*:**:*:*.*:**.:***

MG09190.1 	VYTTRGLIEQGRWALEHAPKVIDYFSEQFEIDYPLPKSDILAVHEF~THGAMENWGLVTY
NCU02319.1	VYTTRGLKEQGRWALEHAPKIIDYFSEQFEIDYPLPKSDILAVHEF~THGAMENWGLVTY
FG01604.1 	VYTTRGLKEQGQWALEHAPKIIDFFSEIFDIDYPLPKSDLIAVHEF~THGAMENWGLVTY
AN4282.1  	VYTTKGLKEQARFALECAHRTVDYFSEIFEIEYPLPKADLLAVHEF0AMGAMENWGLVTY
          	****:** **.::*** * : :*:*** *:*:*****:*::***** : ***********

MG09190.1 	RTTAVLFDEKLSDVRFRNRVAYVVAHE~LAHQWFGNLVTMDW2WDELWLNEGFATWAGWL
NCU02319.1	RMTAILFDEKLSEARFRNRIAYVVAHE~LAHQWFGNLVTMDW~WDELWLNEGFATWAGWL
FG01604.1 	RTTQVLYDEKTSDPRFKNAVAYVVAHE~LAHQWFGNLVTMDW~WDELWLNEGFATWVGWH
AN4282.1  	RTTAVLFDEGKSDTRYKNRIAYVVAHE1LAHQWFGNLVTMDW~WDELWLNEGFATWVGWL
          	* * :*:**  *: *::* :******* ************** *************.** 

MG09190.1 	ATDY1LHPD~WNVWPQFV~NEGMGMAFELDSIRASHPIQVPVRDALDVNQIFDHISYLKG
NCU02319.1	ATDH~LHPD~WEVWPQFI~NEGMDQAFLLDSVRASHPIQVEVRDALDVNQIFDKISYLKG
FG01604.1 	AVDH~LHPD~WQVWAQFV~NEGMEAAFRLDGIRASHPIHVPVRDALDVNQIFDSISYLKG
AN4282.1  	AVDH~FYPE1WNIWSQFV0AEGVQQAFQLDSLRASHPIQVPVKNALEVDQIFDHISYLKG
          	*.*: ::*: *::*.**:  **:  ** **.:******:* *::**:*:**** ******

MG09190.1 	CSVIRMLASHLGVRTFLKGVAMYLKKHQYH~NAKTDALWSALSEASGTDVNALM~APWIE
NCU02319.1	CSMIRMLASHLGIKTFLKGIALYLQRHAYG~NAKTEALWNALSEASGVDVNTIM~RPWIE
FG01604.1 	CSAIRMLANHLGVEVFLKGVSNYLKSHAYG~NAKTTALWDALGEASGKNVTELM~NPWIS
AN4282.1  	SSVIRMLSTHLGRETFLRGVAQYLKTHAYG1NATTNDLWIALSQASNQDVNSFM0DPWIR
          	.* ****:.*** ..**:*:: **: * *  **.*  ** **.:**. :*. :*  *** 

MG09190.1 	KIGHPVLTVTEKSSDGAISVKQSRFLSTGDVKPEDDTTTWWIPLALSGKVGTAEGEVQSL
NCU02319.1	EIGFPVVSVTE--GKDQISVKQARFLSTGDVKPEDDKTTWWVPLSLRGKIGSQD--IEPL
FG01604.1 	KIGHPVLTVAE--EPGQISVKQSRFLSTGDVQPEDDTTTWWVPLGLEGKKDHAG--VASL
AN4282.1  	KIGFPVVTIAE--ETGQLEIRQNRFLSTGDVKPEEDETTWWIPLGIKSGPELTVQ--DPR
          	:**.**::::*    . :.::* ********:**:* ****:**.: .          . 

MG09190.1 	SLTTKEETITGVSDE-FYLLNSNATGFYQVNYPAERLAIFAKQLDRLGPADKIRISSSAS
NCU02319.1	SLQTKETTIDGVSQD-FYQLNANATGFYRVNYPESRLKTLGTQLAHLTTEDKIFITGSAA
FG01604.1 	SLTSKEDTIRDVDED-FYKLNSGATGFYRVNYPPGRLAKLSTQLDKLSTEDKISIIGSTA
AN4282.1  	ALVSKADTIQGIGQDSFYKINKDLSGFYRTNYSSSRLKKLGQSLDSLSTEDKIGLIGDAA
          	:* :*  ** .:.:::** :* . :***:.**.  **  :. .*  * . *** : ..::

MG09190.1 	DLAFSGYAKTSSLLSFLEGFSNETEYL~VLAQALDAVATLKSVFGEDDGIRAGLNAFSLK
NCU02319.1	DLAFAGNSTTAALLSFVQGLKNETHYR~VLSQALDSVNTLKSIFGDDEEVKKGLEKFTLE
FG01604.1 	DLAFAGNGTTPALLTFLEGFGKETHTL~VWRQVLDSISGVKSVFGEDESIKKALDNFTLK
AN4282.1  	ALAVSGESSTAALLSLLEGFKDETNYL2VWSQISSSVGNLRSVFSQNEEVAEGLKKFARE
          	 **.:* ..*.:**::::*: .**.   *  *  .::  ::*:*.::: :  .*. *: :

MG09190.1 	LIENQVDKLSFDVPANDSYTNSLLRKRILLAAVSNGHEG2VTAEAKKRFDAYFNNGDKSA
NCU02319.1	LIDKALKEVGWEPKQGENYNIPLLRKRLLLTAVANSHEE~VINEAFRRWNEWRANPTGAP
FG01604.1 	LINEKVKEVGWEFPEGEDYLTGILRKEIIGVAVASGHSA2VTEEALKRFNAWVEDPEANP
AN4282.1  	LSAPAANMIGWEFKPTDDYLTLQLRKLLIAMSGLAGNES2IVAEAKNRFGLWATAKDKSA
          	*     . :.::    :.*    *** ::  :   .:.  :  ** .*:. :       .

MG09190.1 	VHADLRITCYRAAILSDPATAVQTLKKEWAETKAVDGREVCLGALGHVQDAAIIKDVLLP
NCU02319.1	IPADLRLPVYRAALKKDPANAVAAIKEEWFTTPAIDGKEVCLQALGQVTDETLIEDVLLP
FG01604.1 	IPAPLRVAVWRAAMIKEPARTVEILKKEWLNTKSIDGKLLSLSVLGTVKDAEILKKDVIP
AN4282.1  	IHTNLRSAIFSIAVSEGTREEYDSVKDEYLKTDSVDGKEICLGALGRTKDAALVN-EYLD
          	: : ** . :  *: . .      :*.*:  * ::**: :.* .** . *  :::   : 

MG09190.1 	FLFDASP-----DAVPPADVHIMATALAANRVGRPLLWERLRDDWENSVVKKLGGNPILV
NCU02319.1	FLFDSAPPAHPRDSVPGADMHILSGNMAGNRIARPLLWAYLRDNWD-KFNAKLGGNPILV
FG01604.1 	FNFNQSPPS---NAVPAGDMHVLGGSVANNVVGRPVQWQFMKDNWD-AVITKLG-NPVVV
AN4282.1  	FVFS--------DKVAIQDVHNGAVSLAANSKVRHLLWEYMKNNWD-SVEARLSANNVVF
          	* *.        : *.  *:*  .  :* *   * : *  ::::*:  .  :*..* ::.

MG09190.1 	DRLVKNSLGKFTDAKVVDDITAFFSNQDTSGFNRTLETVKDKVRGRASYKERDAQVLKEW
NCU02319.1	DRMVNVSLPKFADLETLKEIEDFFSKVSTKGFDRTLEQVKDKIRGRAAYKMRDAEVVKSW
FG01604.1 	DRYMNLSLSRFTDVSAVEDIEKFMADKDTSSFNRTLGTVKDKIRGRAAYRERDSEKLKEW
AN4282.1  	ERFVRMGLSKFADHSIGDDIAAFFQDKDTTAYDRALVIVADSIRTSANYKERDEKQVLKW
          	:* :. .* :*:* .  .:*  *: . .*..::*:*  * *.:*  * *: ** : : .*

MG09190.1 	LKENGYLNK
NCU02319.1	LKANGYC--
FG01604.1 	LSAHGYA--
AN4282.1  	LKEHGYA--
          	*. :**
```
